# The Hypoglycemic Effect of the Kelp on Diabetes Mellitus Model Induced by Alloxan in Rats

**DOI:** 10.3390/ijms13033354

**Published:** 2012-03-12

**Authors:** Shao-Hua Long, Zhu-Qin Yu, Li Shuai, Yun-Liang Guo, De-Lin Duan, Xin-Ying Xu, Xiao-Dan Li

**Affiliations:** 1 Institute of Cerebrovascular Diseases, Affiliated Hospital, Qingdao University Medical College, Qingdao Shandong 266003, China; E-Mails: long121400@163.com(S.-H.L.); yuzhuq2008@163.com (Z.-Q.Y.); guoqdsd@163.com (X.-Y.X.); zongshiyi@163.com (X.-D.L.); 2 School of Chemistry, Chemical Engineering and Environmental Sciences, Qingdao University, Qingdao Shandong 266071, China; E-Mail: shuailitunicat@yahoo.com.cn; 3 Institute of Oceanology, Chinese Academy of Sciences, Qingdao 266071, China

**Keywords:** kelp, diabetes mellitus, alloxan, oxidative stress, rats

## Abstract

Hypoglycemic effects and the use of kelp in diabetes mellitus (DM) model rats induced by alloxan were investigated. Sixty healthy male rats were used to establish DM models by injecting alloxan intraperitoneally. Kelp powder was added to the general forage for the rats. The levels of fasting blood glucose (FBG) were determined by an automatic blood glucose device. Electrochemiluminescence immunoassay was applied to determine the serum levels of insulin. The serum levels of malondialdehyde (MDA) were measured by thiobarbituric acid assay and nitric oxide (NO) by nitrate reductase assay. The activities of superoxide dismutase (SOD) were determined by xanthinoxidase assay and glutathione peroxidase (GSH-Px) by chemical colorimetry. The shape and structure of islet cells were observed with Hematine-Eosin staining, and the expression of superoxide dismutase (SOD) and inducible nitric oxide synthase (iNOS) in islet cells were detected by immunohistochemical assay. The results showed that the serum levels of insulin after treatment with kelp powder increased significantly compared to those in the DM-model group, while the FBG in the medium-high dose treated groups decreased significantly compared to those in the DM-model group (*P* < 0.05). The levels of MDA and NO in the kelp powder groups were lower than those in the DM-model group, while the activities of SOD and GSH-Px were higher than those in the DM-model group, of which a significant difference existed between the medium-high dose treated groups and the DM-model group (*P* < 0.05). The shape and structure of islet cells improved with the up-expressing SOD and down-expressing iNOS in the medium-high dose treated groups compared to those in the DM-model group (*P* < 0.05). There were no significant differences between the medium and high dose treated groups, all above indexes (*P* > 0.05). It is suggested that kelp might aid recovery of the the islet cell secreting function and reduce the level of FBG by an antioxidant effect.

## 1. Introduction

Diabetes mellitus (DM) is a common disease in the world and type 2 DM accounts for about 90%–95% of cases. Although the etiology of type 2 DM is complex and its pathogenesis is not completely understood, the disease is associated with the level of free radicals and the antioxidant system dysfunction. It has been well established that oxidative stress is a commonly used approach to DM and its complications [1]. Malondialdehyde (MDA) can reflect oxygen free radicals (OFR) in the body that are produced when OFR oxidizes polyunsaturated fatty acids in bio-membranes [2]. The gas free radical, nitric oxide (NO), is important in initiating type 2 DM, insulin resistance and secondary effects as well as the islet beta cell function obstacle [3,4]. The natural enzyme superoxide dismutase (SOD) can capture free radicals in the body and the catalytic oxidation enzyme glutathione peroxidase (GSH-Px) is widespread in the body. These enzymes work to eliminate OFR in the body when decline in activity leads to OFR accumulation [5,6].

*Laminaria japonica* (*L. japonica*) is a widely cultivated kelp, with China being the largest producer [7]. In China, the kelp is used for food in daily life and also used in traditional medicine. According to the “Compendium of Materia Medica” [8], kelp is cold, salty, has efficacy in clearing water, is soft, firm, dissipating and can dissolve phlegm [9], as well as alleviate edema, and eliminate carbuncle. Kelp belongs to the *Phaeophyta Laminariaceae Laminaria*, containing laminarin, ammonium alginate, mannitol, vitamins, amino acids and various normal and trace elements, with a variety of 40 active components [10]. The variety of physiological functions of the kelp relate to the biological activity of polysaccharides which can improve the immunity function, anti-aging, anti-tumor [11], anti-atheroscloresis, anti-diabetics [12,13] and other such biological activity. Until now, there has been only limited research and reports on *L. japonica’s* anti-diabetic functions and mechanism [14]. Therefore, the experiment described in this paper expands the exploration of the hypoglycemic effect along with a possible mechanism of the effect of kelp on alloxan-induced diabetic rats.

## 2. Results and Discussion

### 2.1. General Situation

Before alloxan was injected, all rats reacted nimbly, had hair that was bright and smooth and there was no significant variation in body weights (*F* = 0.05, *q* = 0.03–0.55, *P* > 0.05). After alloxan was injected and before kelp powder, forage was administered, and the animals showed typical signs of diabetes mellitus: clumsiness, slow actions, dull colored fur and marasmus. Average body weights reduced significantly before alloxan was injected (*F* = 1643.22, *q* = 21.77–104.53, *P* < 0.05) and there was no significant difference between the weights of kelp-treated rats and DM-model rats (*P* > 0.05).

After kelp powder forage was administered, the action and hair color of animals in kelp treated and DM-model groups recovered gradually, with body weight becoming significantly higher than before treatment, lower than that of the control group (*F* = 149.29, *q* = 22.82–29.00, *P <* 0.05). Average body weight of animals in the DM-model and kelp-treated groups was significant lower than that in the control group (*F* = 149.29, *q* = 22.82–30.06, *P <* 0.05). There was no significant difference between the low-dose group and DM-model rats (*P* > 0.05). Average body weight of animals in the medium-dose and high-dose groups was significantly higher than in the DM-model rats (*P* < 0.05), but there was no significant difference between medium-dose and high-dose groups (*P* > 0.05) (Table 1).

### 2.2. The Level of Fasting Blood Glucose (FBG)

Before injecting alloxan, there were no obvious differences in FBG levels among the control group, DM-model rats and kelp-treated groups (*F* = 0.05, *q* = 0.03–0.55, *P* > 0.05). After injecting alloxan and before administering kelp powder forage, FBG levels increased significantly compared to those of DM-model rats and the control group (*F* = 79.52, *q* = 19.68–20.19, *P* < 0.05). There was no significant difference between kelp-treated groups and DM-model rats (*P* > 0.05).

After administering kelp powder forage, FBG levels in kelp-treated groups were significantly lower than in the DM-model rats and higher than in the control group (*F* = 189.19, *q* = 9.24–36.44, *P <* 0.05). After the experiment, FBG levels in the DM-model rats and kelp-treated groups were significantly higher than in the control group (*F* = 188.99, *q* = 9.20–36.41, *P* < 0.05). There was no significant difference between the low-dose group and DM-model rats (*P* > 0.05). FBG levels in the medium-dose group and high-dose group were significantly lower than in the DM-model rats (*P* < 0.05) but there was no significant difference between the medium-dose group and the high-dose group (*P* > 0.05). Results indicated that medium-dose kelp could achieve an ideal hypoglycemic effect (Table 2).

### 2.3. The Serum Level of Insulin

The serum levels of insulin in the DM-model group (11.23 ± 3.45, pmol/L) were significantly lower than in the control group (71.38 ± 15.26, pmol/L), while those in kelp-treated groups were significantly higher than those in the DM-model group (*F* = 13.250, *q* = 5.12–9.73, *P* < 0.05), but there were no significant differences between high-dose (26.22 ± 4.85, pmol/L), medium-dose (24.17 ± 5.09, pmol/L) and low-dose (18.78 ± 4.56, pmol/L) groups (*P* > 0.05).

### 2.4. The Levels of MDA and NO and the Activities of SOD and GSH-Px

Serum levels of MDA and NO were significantly higher and actions of SOD and GSH-Px were sharply lower in the DM-model rats and kelp-treaded groups than in the control group (*F* = 12.60, *q* = 3.72–7.17, *P* < 0.05). MDA and NO serum levels were significantly lower and actions of SOD and GSH-PX were higher in medium-dose and high-dose groups than in DM-model groups (*P* < 0.05). There was no significant difference between medium-dose and high-dose groups (*P* > 0.05). Results indicated that medium-dose kelp could achieve ideal anti-oxidant effect (Table 3).

### 2.5. Pancreatic Islets Tissue Pathology

**Figure 1:**
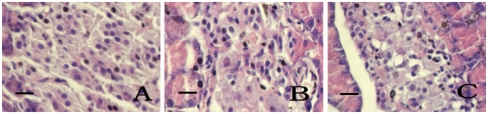
The comparison of the pancreatic islet cells. HE × 400, bar 25 μm. (**A**) Control group, (**B**) DM-model group, and (**C**) Medium-dose kelp-treated group.

The cells of pancreatic islets in the control group are oval in shape, uniform in size and evenly dispersed in the pancreatic gland bubble. DM-model rats have shrunken islets, a reduced number of islet cells that are unevenly dispersed, vacuolar degeneration and karyolysis, etc. but this improved significantly in medium-dose and high-dose kelp-treated groups. The index of pancreatic B cells in medium-dose and high-doses kelp-treated groups is higher than in the DM-model rats (Table 4 and Figure 1).

### 2.6. Immunohistochemistry of SOD and iNOS

**Figure 2:**
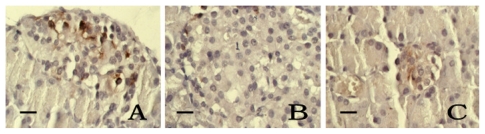
The expression of inducible nitric oxide synthase (iNOS) in the pancreatic islet cells, SABC × 400, bar 25 μm. (**A**) Control group, (**B**) DM-model group, and (**C**) Medium-dose kelp-treated group.

SOD expression in pancreatic islet cells of control group rats was strong and scattered focally with deep-yellow color. SOD expression was reduced in DM-model rats (*t* = 7.89, *P* < 0.05). In medium-dose and high-dose kelp-treated group rats, SOD expression in islet cells was stronger than that in DM-model rats (*t* = 4.73–4.76, *P* < 0.05). There was no significant difference between the low-dose kelp-treated group and the DM-model rats (*t* = 1.69, *P* > 0.05). (Table 4 and Figure 2).

**Figure 3:**
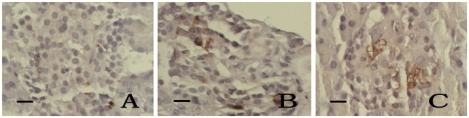
The expression of superoxide dismutase (SOD) in the pancreatic islet cells, SABC × 400, bar 25 μm. (**A**) Control group, (**B**) DM-model group, and (**C**) Medium-dose kelp-treated group.

iNOS expression in pancreatic islet cells of control group rats was weak and scattered focally with light-yellow color and was significantly stronger in DM-model rats (*t* = 8.16, *P* < 0.05). In medium-dose and high-dose kelp treated group rats, iNOS expression in islet cells was weaker than in the DM-model rats (*t* = 4.81–5.30, *P* < 0.05). There was no significant difference between the low-dose kelp treated group and the DM-model rats (*t* = 1.90, *P* > 0.05). (Table 4 and Figure 3).

### 2.7. Discussion

Hyperglycemia and oxidative stress are closely related to diabetes mellitus and its complications. The “common soil” theory of Ceriello *et al.* [15,16] holds that oxidative stress is a common feature of insulin resistance, diabetes mellitus and cerebrovascular diseases [17]. In a body suffering from various harmful stimuli, there is an increase in free radicals and decreased elimination of those radicals can upset the balance of the oxidation system and antioxidant system, and damage tissues and function [18,19]. At the same time, an overflow of oxygen free radicals (OFR) causes lipid peroxidation (LPO), which increases the damage of the oxidative stress through a chain reaction [20,21]. Diabetes mellitus patients with long-term hyperglycemia produce more OFR due to increased glucose autoxidation and protein saccharification, which weakens oxidation resistance and initiates oxidation stress [22]. Increased OFR plays an important role in initiating type 2 diabetes mellitus, insulin resistance and secondary effects, and the islet beta cell function obstacle. It becomes an important media for various factors that cause type 2 diabetes mellitus [23].

Alloxan has a specific toxic effect on islet beta cells that can cause damage by producing a superoxide radical, damage the DNA of the cell and activate polyphosphate ADP ribosomes synthase. This can reduce the coenzyme I, impair mRNA function and cause proinsulin decrease and insulin shortage [24]. In the process of diabetes mellitus development, the anti-oxidant defense level drops and the ability to eliminate free radicals weakens [25,26]. Bottino *et al*. [27] separated and purified islet cells by using an anti-oxidant to disrupt islet cells at an early stage and found that if blocking oxidative stress reduces damage to the islet cells and promotes their proliferation, it provides a new way of thinking about early diagnosis and intervention for diabetes mellitus. Our previous researches confirmed that kelp has antioxidant effects on the organism and can improve the oxidative stress [12,13].

In this experiment, the serum MDA and NO content in DM-model rats were significantly higher than those in the control group. The action of serum SOD and GSH-Px in DM-model rats was significantly less than in the control group. At the same time, SOD expression in pancreatic islet cells in the DM-model rats was significantly lower and iNOS expression was higher than in the control group. The number of islet cells and beta cells reduced significantly and become partly pyknotic and there was necrosis related to a decrease in serum insulin levels as well as increases in fasting blood glucose levels. Such features indicate that diabetes mellitus models, induced by alloxan, caused much lipid peroxidation in the rat body, injured the structure and secretion function of the islet cells and increased fasting blood glucose levels.

After kelp powder was added to interfere with the diabetes mellitus models induced by alloxan, serum levels of MDA and NO decreased significantly and the actions of serum SOD and GSH-Px increased in comparison with the DM-model group. Immunochemical stain showed that SOD expression in pancreatic islet cells was significantly higher and iNOS expression was lower than in the DM-model group. The structure of the pancreatic islet cells in kelp powder treated groups clearly improved in comparison with the DM-model group. Apparently, kelp enhances anti-oxidant enzyme activity, reduces lipid peroxidation (LPO) and products induced by diabetes mellitus and shows an antioxidant effect in the organism. Changes in fasting blood glucose (FBG) levels, serum insulin levels and animal weights show that FBG levels in kelp powder treated groups are significantly lower than in the DM-model group, while serum insulin levels and animal weights were higher than in the DM-model group. Results indicate that kelp could play a hypoglycemic role by enhancing anti-oxidation and enabling recovery of the pancreatic islet cell secreting function.

## 3. Experimental Section

### 3.1. The Creation of Diabetic Models

Sixty SPF grade healthy male *Wistar* rats weighing 140–160 g were purchased from the Experiment Animal Center of Qingdao Drug Inspection Institute (SCXK (LU) 20090100). Local regulations related to ethical experimentation on animals and guidelines for the care and use of laboratory animals were followed in all animal procedures in this experiment. This experiment was approved by the Ethics Committee of Qingdao University Medical College (No. QUMC 2011-09). Animals were acclimatized for 7 days and allowed free access to food and water at room temperature (23 ± 2 °C) and humidity-controlled housing with natural illumination. Initially, an extracted blood sample (0.5 mL) from the tail vein was used for the serum separation and determination of fasting blood glucose (FBG) levels. Subsequently, ten (n = 10) experimental animals were randomized as a control group and injected with equivalent normal saline and the remaining 50 rats were injected intraperitoneally (i.p.) with 1.5% alloxan (100 mg/kg body weight), once every three days and three times continuously [28,29]. Three days after the final injection, the FBG level was determined and FBG > 15.00 mmol/L served as the standard in successful diabetes mellitus (DM) models. Ten of the 50 experimental rats were excluded because they did not satisfy the standard. The remaining 40 DM-model rats were subjected to experiment and randomly divided into a DM-model group (n = 10), and three kelp treated groups: a low-dose group (1.25 g/kg, n = 10), a medium-dose group (5.0 g/kg, n = 10) and a high-dose group (12.5 g/kg, n = 10).

### 3.1. The General Forage and Kelp Powder Forage

The main components of the general forage: soybean meal 20.4 %, corn flour 31.7%, wheat bran 7.2%, wheat flour 28.8%, fish meal 7.2%, yeast 2.4%, salt 0.2%, bone meal 1.4%, cod liver oil 0.04%, Vitamin E powder 0.04%, milk powder 0.18%, trace elements 0.04%.

“Zhongke No.1” kelp powder forage is derived from kelp products manufactured at the Institute of Oceanology, Chinese Academy of Sciences. Chemical analysis shows the main components to be dietary fiber 26.1%, protein 8.5%, lipid 0.39%, total amino acid 10.49 mg/100g, Vitamin A 273 μg/100g, and Vitamin C 3 μg/100g.

The kelp powder forage used as an ingredient in the feed for the rats was pressed into a block, air dried and reserved. The low-dose forage contains general forage 97.5% and kelp powder 2.5%, the medium-dose forage contains general forage 90% and kelp powder 10%, and the high-dose forage contains general forage 75% and kelp powder 25%.

### 3.2. Inference of Tests

Rats in the control group and DM-model group were fed with general forage for two weeks. Rats in the kelp-treated groups were fed with kelp powder forage for two weeks.

The low-dose kelp powder (2.5%) equals 1.25g/kg body weight per day, the medium-does kelp powder (10%) equals 5.0 g/kg body weight per day and the high-dose kelp powder (25%) equals 12.5 g/kg body weight per day.

### 3.3. Preparation of Samples

#### 3.3.1. Serum preparation

At the end of this experiment, all rats were denied food for 12 h, then FBG was determined and 4 mL blood was collected from the eye artery using heparinized capillary tubes. Blood samples were centrifuged for 10 minutes at 4000 r/min to separate serum and then stored at −20 °C.

#### 3.3.2. Pancreatic tissue

At the end of this experiment, animals were sacrificed by cervical dislocation and pancreatic tissues were immediately collected. Rudimental blood was fully washed with normal saline at −4 °C, and placed in 4% formaldehyde for fixing.

### 3.4. Index of Determinations

#### 3.4.1. FBG Level

An automatic blood glucose meter (Johnson & Johnson Medical Equipment Co., Ltd., Germany) and blood glucose test strips (Onetouch, Ultra) were used to detect FBG level (mmol/L).

#### 3.4.2. The Serum Level of Insulin

Serum samples were thawed at room temperature and centrifuged again. Electrochemiluminescence immunoassay (ECLIA) was applied to determine the serum level of insulin with Elecsys 2010 and Cobase 411 analyzers and Roche diagnostics reagent kits (12017547). All standards were prepared before starting the assay procedure. The first incubation: insulin from 20 μL serum sample, a biotinylated monoclonal insulin-specific antibody, and a monoclonal insulin-specific antibody labeled with a ruthenium complex form a sandwich complex. The second incubation: after addition of streptavidin-coated microparticles, the complex becomes bound to the solid phase via interaction of bitin and strepavidin. Then the reaction mixture is aspirated into the measuring cell where the microparticles are magnetically captured onto the surface of the electrode. Unbound substances are then removed with ProCell. A voltage is applied to the electrode to induce chemiluminescent emission which is measured by a photomultiplier. The results are determined via a calibration curve which is instrument-specifically generated by 2-point calibration and a master curve provided via the reagent barcode. Finally, the analyzer automatically calculates the analyte concentration of each sample. The measuring range of the assay is 1.39–6945 pmol/L.

#### 3.4.3. MDA and NO Values

MDA values were detected by thiobarbituric acid and NO values were detected by nitratase reductase using kits purchased from Jiancheng Institute of Biomedical Technology, Nanjing China. Standardization was conducted on an ultraviolet spectrophotometer (Bechmann DU640, USA) and the selected wavelengths were 532 nm for MDA and 550 nm for NO. Assay sensitivity is 0.1 mmol/L (MDA) and 0.1 μmol/L (NO).

#### 3.4.4. SOD and GSH-Px Activities

SOD action was detected by xanthinoxidase and GSH-Px action was detected by chemical colorimetry with kits supplied by Jiancheng Institute of Biomedical Technology, Nanjing China. Standardization was conducted on an ultraviolet spectrophotometer and the selected wavelengths were 550 nm for SOD and 412 nm for GSH-Px. Assay sensitivity is 0.1 U/mL (SOD and GSH-Px).

#### 3.4.5. Histopathological Assay

Pancreatic tissue samples fixed in 4% formaldehyde were gradually dehydrated in alcohol, hyalinized by dimethylbenzene, embedded in paraffin, sectioned at a thickness of 5 μm, adhered to sections prepared with poly-L-Lysine and stored at 4 °C. Paraffin sections were deparaffinaged deparaffinated by dimethylbenzene, hydrated in gradient ethanol washed with distilled water and stained by Hematine-Eosin (HE) staining, that showed nuclei with a blue color and cytoplasm with a red color under light microscopy. Under a 400-fold light microscope, the average index of pancreatic B cells was calculated in five views selected randomly in each section from each animal. Index of pancreatic B cell in each view = (the number of pancreatic islets B cells/total number of cells) × 100.

#### 3.4.6. Immunohistochemical Assay

Rabbit anti-rat SOD and iNOS multi-clonal antibody, and strept actividin-biotin peroxidase complex (SABC) kit, diaminobenzidine (DAB) kit were supplied by Wuhan Boster Biological Technology Co. Ltd. China. Paraffin-embedded sections were deparaffinaged in dimethylbenzene, hydrated successively in gradient ethanol and antigen was restored twice in a microwave oven. All procedures were strictly performed in accordance with the manufacturer’s directions. Under a microscope, cells with brown granulation in cytoplasm or nucleus were considered to be positive. Negative control slides added 0.01 mol/L PBS (containing 1:200 blocking serum of non-immunized animals) instead of a primary antibody that has no immunological reaction. Under a 400× light microscope, five sections were randomly chosen from each experimental rat and observed in five views detected in islets. Absorbance values (*A*) of each view were detected by a LEICA Qwin microgramme analytical system (Leica Company, Shanghai, China).

### 3.5. Statistical Analysis

SPSS17.0 software was used for statistical analysis. Data were expressed as mean ± standard deviation (*χ̄* ± s). Multi-group comparison was made by analysis of variance (ANOVA) and Student’s test and two-group comparison was by *t*-test. Values were considered to be significant when *P* < 0.05.

## 4. Conclusions

This study suggested that kelp might enable the recovery of islet cell secretion function and reduce the FBG level by an antioxidant effect. Future research should focus on the hypoglycemic effect and the mechanism of kelp.

## Figures and Tables

**Table 1:** The average body weights of animals in the experiment (*χ̄* ± s, Unit: g).

Groups	n	Dose	Before experiment	Before kelp-treated	After kelp-treated
Control group	10	General forage	151.76 ± 3.45	168.50 ± 4.22	189.69 ± 4.55
DM-model group	10	General forage	151.85 ± 3.67	133.62 ± 5.35 a	139.46 ± 5.36
Low-dose group	10	1.25 g/kg kelp	151.68 ± 3.38	134.46 ± 5.23 a	141.24 ± 5.32 bc
Medium-dose group	10	5.0 g/kg kelp	152.26 ± 3.51	135.27 ± 5.18 a	150.24 ± 5.45 b,c,d
High-dose group	10	12.5 g/kg kelp	151.65 ± 3.43	133.55 ± 5.27 a	151.56 ± 5.67 b,c,d

a *P* < 0.05 *vs.* before experiment;

b *P* < 0.05 *vs.* before kelp-treated;

c *P* < 0.05 *vs.* DM-model group;

d *P* < 0.05 *vs.* low-dose group.

**Table 2:** The levels of fasting blood glucose (FBG) in the experiment (*χ̄* ± s, mmol/L).

Groups	n	Dose	Before experiment	Before kelp-treated	After kelp-treated
Control group	10	General forage	4.78 ± 0.39	4.95 ± 0.34	4.97 ± 0.33
DM-model group	10	General forage	4.82 ± 0.33	17.86 ± 2.26 a	13.32 ± 1.40 b
Low-dose group	10	1.25 g/kg kelp	4.55 ± 0.35	18.12 ± 2.28 a	12.63 ± 1.67 b
Medium-dose group	10	5.0 g/kg kelp	4.81 ± 0.37	17.79 ± 2.31 a	9.37 ± 1.70^b^,^c^,^d^
High-dose group	10	12.5 g/kg kelp	4.65 ± 0.34	18.05 ± 2.35 a	9.18 ± 1.65^b^,^c^,^d^

a *P* < 0.05 *vs.* before experiment;

b *P* < 0.05 *vs.* before kelp-treated;

c *P* < 0.05 *vs.* DM-model group;

d *P* < 0.05 *vs.* low-dose group.

**Table 3:** The levels of malondialdehyde (MDA) and nitric oxide (NO) and the activities of superoxide dismutase (SOD) and glutathione peroxidase (GSH-Px) in the experiment (*χ̄* ± s).

Groups	n	Dose	MDA(mmol/L)	NO(μmol/L)	SOD(U/mL)	GSH-Px(U/mL)
Control group	10	General forage	7.15 ± 0.68	14.96 ± 1.56	156 ± 14.02	922 ± 22.16
DM-model group	10	General forage	9.38 ± 1.24^a^	23.86 ± 2.17 a	122 ± 11.26 a	828 ± 15.46 a
Low-dose group	10	1.25 g/kg kelp	8.93 ± 1.02	21.50 ± 2.24	127 ± 18.35	837 ± 24.82
Medium-dose group	10	5.0 g/kg kelp	8.02 ± 0.45 b,c	17.13 ± 1.41 b,c	143 ± 22.26 b,c	890 ± 24.58 b,c
High-dose group	10	12.5 g/kg kelp	7.83 ± 0.51^b^,^c^	16.32 ± 1.73 b,c	145 ± 19.38 b,c	886 ± 25.72 b,c

a *P* < 0.05 vsrsus control group;

b *P* < 0.05 versus DM-model group;

c *P* < 0.05 versus low-dose group.

**Table 4:** The B cell index and the expressions of SOD and inducible nitric oxide synthase (iNOS) in the panreatic tissue (*χ̄* ± s).

Groups	n	Dose	B cell index(%)	SOD (A)	iNOS(A)
Control group	10	General forage	61.48 ± 9.13	0.48 ± 0.15	0.16 ± 0.05
DM-model group	10	General forage	28.16 ± 5.64 a	0.41 ± 0.12 a	0.41 ± 0.12 a
Low-dose group	10	1.25g/kg kelp	31.49 ± 6.28	0.22 ± 0.08	0.35 ± 0.10
Medium-dose group	10	5.0g/kg kelp	45.37 ± 6.82 b	0.31 ± 0.10 b	0.24 ± 0.09 b
High-dose group	10	12.5g/kg kelp	46.71 ± 7.36 b	0.33 ± 0.12 b	0.23 ± 0.08 b

a *P* < 0.05 versus control group;

b *P* < 0.05 versus DM-model group.
